# Unveiling the impact of lipid metabolism on triple-negative breast cancer growth and treatment options

**DOI:** 10.3389/fonc.2025.1579423

**Published:** 2025-05-29

**Authors:** Xin-xian Cai, Zhe-zhong Zhang, Xiao-xiao Yang, Wen-rui Shen, Liu-wei Yuan, Xi Ding, Ying Yu, Wen-yu Cai

**Affiliations:** ^1^ The First Affiliated Hospital of Zhejiang Chinese Medical University (Zhejiang Provincial Hospital of Chinese Medicine), Hangzhou, China; ^2^ School of Medical Technology and Information Engineering, Zhejiang Chinese Medical University, Hangzhou, China; ^3^ The First Clinical Medical College, Zhejiang Chinese Medical University, Hangzhou, China

**Keywords:** triple-negative breast cancer, lipid metabolism, energy metabolism, recurrence of metastases, cell death

## Abstract

Triple-negative breast cancer (TNBC) is a subtype of breast cancer associated with poor prognosis and limited targeted treatment options. Lipid metabolism plays a pivotal role in the initiation, progression, and metastasis of TNBC by supporting cancer cell energy production, facilitating membrane biosynthesis, and regulating signal transduction. Dysregulation of lipid metabolism promotes tumor cell proliferation and contributes to processes such as epithelial-mesenchymal transition (EMT), angiogenesis, and immune evasion. Targeting lipid metabolism—such as inhibiting fatty acid synthase (FASN) and lipid metabolic byproducts—has emerged as a promising therapeutic strategy. The integration of multi-omics approaches and advanced imaging technologies can further elucidate the interactions between lipid metabolism and the tumor microenvironment, thereby supporting precision oncology. Future research should explore the role of lipid metabolism in distinct TNBC subtypes, optimize therapeutic strategies, and improve patient outcomes, particularly for those who are unresponsive to conventional treatments.

## 1 Introduction

Triple-negative breast cancer (TNBC) is a distinct subtype of breast cancer, accounting for approximately 24% of all cases. It is characterized by the absence of estrogen receptor (ER), progesterone receptor (PR), and human epidermal growth factor receptor 2 (HER2) expression, rendering it unresponsive to conventional hormone and targeted therapies (1). The high heterogeneity of TNBC poses significant challenges for traditional molecular classification methods, which often fail to fully capture its complex biological characteristics. To address this limitation, recent studies have shifted focus toward metabolic reclassification of TNBC, aiming to elucidate its underlying metabolic heterogeneity and inform precision treatment strategies. Lehmann et al. pioneered this effort by subdividing TNBC into four subtypes—basal-like 1 (BL1), basal-like 2 (BL2), mesenchymal (M), and luminal androgen receptor (LAR)—based on gene expression profiles and the influence of tumor-infiltrating lymphocytes and tumor-associated stromal cells (2). Building on this, Gong et al. (3) introduced the metabolism-pathway-based subtypes (MPSs) classification system, which integrates transcriptomic and metabolic features to categorize TNBC into three distinct subtypes: fatty acid synthesis (MPS1), glycolysis (MPS2), and mixed (MPS3). Comparative analysis of these classifications reveals that MPS1 predominantly aligns with the LAR subtype, while MPS2 is characterized by a high prevalence of basal-like features. In line with its mixed phenotype, MPS3 comprises tumors exhibiting diverse molecular subtypes. Due to the lack of effective therapeutic targets, patients with TNBC often experience poor prognoses and a heightened risk of metastasis and recurrence. In recent years, tumor metabolic reprogramming has emerged as a key driver of cancer progression (4), with lipid metabolism becoming a critical aspect of cancer cell metabolism. Lipid metabolism plays a fundamental role in sustaining energy production, facilitating membrane biosynthesis, and regulating signal transduction, while being intricately linked to cancer initiation, progression, metastasis, and recurrence (5).

In TNBC, dysregulated lipid metabolism contributes to the rapid proliferation of cancer cells and their adaptation to the tumor microenvironment. Studies have demonstrated that TNBC cells modulate fatty acid, cholesterol, and triglyceride metabolism not only to meet their high energy demands but also to promote proliferation, migration, and drug resistance (6). Furthermore, lipid metabolic byproducts actively shape the tumor microenvironment by promoting angiogenesis, cell migration, and epithelial-mesenchymal transition (EMT), thereby accelerating metastasis and recurrence (7). Additionally, abnormal lipid metabolism has been implicated in interactions with various cell death pathways, including ferroptosis and apoptosis, further influencing the balance between TNBC cell survival and death (8, 9).Recently, therapeutic strategies targeting lipid metabolism have garnered increasing interest. These approaches include interventions aimed at key metabolic enzymes, lipid-derived metabolites, and the interplay between lipid metabolism and the immune microenvironment. Given the distinct lipid metabolism profiles across TNBC subtypes, it is crucial to account for the metabolic characteristics of these subtypes when developing treatment strategies to ensure targeted and effective therapeutic outcomes. Such strategies offer promising avenues for novel TNBC treatment modalities and improved patient outcomes.

This review aims to comprehensively summarize the fundamental mechanisms of lipid metabolism in TNBC, explore its role in tumor metastasis, recurrence, cell death, and immune modulation, and integrate current clinical research advancements. By doing so, we aim to provide a theoretical framework and practical insights for the future development of lipid metabolism-targeted therapies.

## 2 Fundamental mechanisms of lipid metabolism in TNBC

### 2.1 Lipid metabolic pathways and energy metabolism in cancer cells

**Figure 1:**
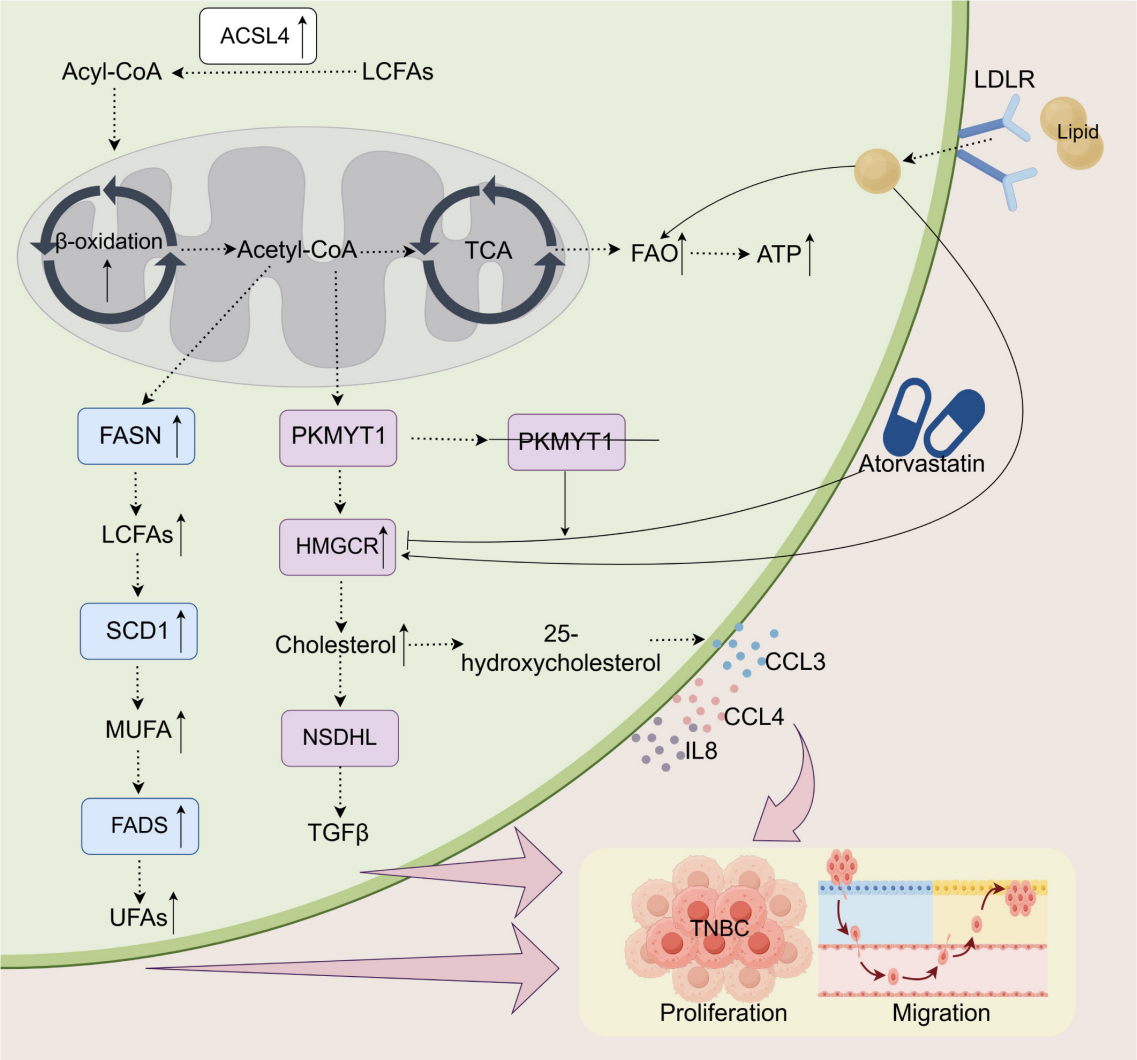
Lipid metabolism and its impact on the proliferation and migration of TNBC. In TNBC, alterations in lipid metabolism play a crucial role in supporting tumor progression. Enhanced FAO, through an upregulated β-oxidation process, provides ATP for tumor cells and is modulated by key enzymes such as FASN, SCD1, PKMYT1, and ACSL4. The metabolism of long-chain and unsaturated fatty acids is intensified, generating essential metabolites like acetyl-CoA and cholesterol, which facilitate both cell proliferation and migration. LDLR promotes cholesterol uptake, while 25-hydroxycholesterol stimulates the secretion of immune-regulatory factors, including CCL3, CCL4, and IL-8. PKMYT1 knockdown increases sensitivity to atorvastatin, which, by inhibiting cholesterol synthesis, reduces tumor growth and metastasis. These metabolic changes enhance the adaptability of tumor cells, further promoting the progression of TNBC.

Lipid metabolism is crucial for TNBC cells, particularly in the regulation of fatty acid and cholesterol metabolism. These metabolic pathways supply TNBC cells with the necessary energy and structural components for membrane biosynthesis, thereby facilitating their rapid proliferation, migration, and resistance to therapeutic interventions (**Figure 1**). TNBC cells demonstrate metabolic plasticity to overcome nutrient scarcity, acquiring stromal-derived fuels to sustain proliferation, drive chemoresistance, and remodel the immunosuppressive microenvironment (10).

#### 2.1.1 Fatty acid metabolism

Fatty acids are essential components of cell membranes and play critical roles in signal transduction, energy metabolism, and biomolecule synthesis. In TNBC, fatty acid metabolism is frequently reprogrammed to support cancer cell proliferation, survival, and metastasis. Notably, lipid synthesis, particularly the fatty acid synthesis pathway, is often upregulated, primarily driven by the activation of fatty acid synthase (FASN). FASN, a key enzyme in fatty acid biosynthesis (11), is overexpressed in TNBC cells and is strongly associated with tumor aggressiveness and metastatic potential (12, 13). CPT1-mediated fatty acid β-oxidation critically promotes TNBC metastasis. Pharmacological CPT1 inhibition disrupts mitochondrial function through membrane potential loss, ROS accumulation, and ATP depletion, ultimately suppressing tumor growth and inducing apoptosis (14). This highlights CPT1 as a potential therapeutic target for TNBC. Furthermore, alterations in fatty acid metabolism influence intricate cross-regulatory networks across multiple critical signaling pathways. The overexpression of FASN in TNBC not only directly enhances fatty acid synthesis but also activates mechanistic target of rapamycin complex 1 (mTORC1) signaling, which induces sterol regulatory element-binding protein (SREBP)-mediated lipid synthesis. This process supports oncogene-driven mechanisms, ultimately facilitating the abnormal growth and proliferation of TNBC cells (15). Abnormal lipid metabolism in tumor cells is characterized by an increased rate of fatty acid β-oxidation, generating additional energy for the cells. This metabolic shift allows tumor cells to adapt to hypoxic and nutrient-deprived environments, thus facilitating tumor growth and metastasis. Studies have shown that TNBC cells cultured in adipose tissue-conditioned medium exhibit significantly elevated β-oxidation levels (16). Furthermore, TNBC cells regulate lipoprotein synthesis and secretion, altering lipid distribution and metabolism to enhance their adaptation to nutrient and oxygen deprivation. Certain fatty acids may also act as signaling molecules, directly modulating oncogenic pathways. These metabolic alterations create a microenvironment conducive to TNBC progression, enabling tumor cells to survive and proliferate. Additionally, nitro-fatty acid derivatives, such as NO2-OA, have been shown to suppress TNBC cell proliferation and viability by inhibiting TNF-α signaling, thereby reducing TNBC cell migration and invasion (17). This finding holds significant therapeutic implications for TNBC and offers new potential treatment strategies.

TNBC cells modulate pathways such as fatty acid synthesis and β-oxidation to generate essential lipid molecules and energy required for their proliferation and survival. Therefore, targeting fatty acid metabolism represents a promising therapeutic strategy for TNBC.

#### 2.1.2 Cholesterol metabolism

Cholesterol is a fundamental component of cell membranes and plays a crucial role in hormone synthesis, signal transduction, and energy metabolism. In TNBC, cholesterol synthesis is frequently upregulated to meet the proliferative demands of cancer cells, a process regulated by key enzymes such as HMG-CoA reductase (HMGCR). HMGCR, the rate-limiting enzyme in cholesterol synthesis, serves as a target for antihypercholesterolemic drugs (18). Cholesterol contributes to cell membrane proliferation and tumor cell migration (19), and its accumulation in mammary adipose tissue may enhance the aggressiveness of breast cancer, particularly in multifocal TNBC (20). Furthermore, the cholesterol metabolism enzyme NSDHL drives the proliferation and migration of TNBC cells by activating the TGFβ signaling pathway (21). While atorvastatin inhibits cholesterol synthesis by targeting HMGCR, it feedback-activates SREBF2, resulting in the upregulation of cholesterol synthesis. Protein Kinase, Membrane Associated Tyrosine/Threonine 1 (PKMYT1), a key regulator of cholesterol synthesis in TNBC cells, plays a critical role in this process; its knockout suppresses this feedback activation, thereby potentiating the antitumor efficacy of statins (22). Collectively, cholesterol synthesis is integral to the malignant phenotype of TNBC and offers genetic insights into the potential risks associated with lipid-lowering therapies. In addition to synthesis, cholesterol metabolism also involves the regulation of cholesterol uptake, which supports tumor growth. A study by O’Neill K (23) revealed that TNBC cells rely on exogenous cholesterol acquisition to maintain their viability and invasive phenotype. Increased cholesterol uptake further enhances TNBC metabolic pathways, including oxidative phosphorylation, tricarboxylic acid (TCA) cycle activity, and aerobic glycolysis (24).Moreover, cholesterol-derived metabolites, such as steroid and adrenal hormones, contribute to tumor progression by acting as signaling molecules that promote cancer cell proliferation and survival (19). For example, 25-hydroxycholesterol has been found to induce the secretion of chemokines IL-8, CCL3, and CCL4, thus promoting TNBC cell migration (25). Additionally, cholesterol modulates TNBC metabolism by influencing key transcription factors. Cholesterol plays a role in TNBC metabolism by modulating key transcription factors, including RORβ. Inhibition of cholesterol reduces both its content and synthesis rate in tumors, highlighting its regulatory significance in TNBC progression (26).

Cholesterol metabolism plays a crucial role in the pathogenesis of breast cancer, involving multiple key enzymes, transcription factors, and metabolic pathways. These findings provide a solid theoretical foundation for the clinical evaluation of cholesterol-targeting therapies, which may offer promising new strategies for breast cancer treatment.

### 2.2 The interplay between mitochondrial energy metabolism and lipid metabolism

Mitochondria are the primary energy-generating organelles in cells, producing ATP through oxidative phosphorylation to meet cellular energy demands. Recent studies have shown that cancer cells, particularly those of TNBC, undergo extensive metabolic reprogramming, enabling them to dynamically adjust metabolic pathways in response to environmental fluctuations. Among these pathways, lipid metabolism plays a central role in maintaining TNBC energy homeostasis (10). TNBC cells demonstrate significant metabolic plasticity, dynamically shifting between aerobic glycolysis and oxidative phosphorylation in response to fluctuating microenvironmental conditions. This adaptive capability is modulated by mitochondrial dynamics, which regulate both the structural integrity and functional efficiency of mitochondrial networks. Specifically, mitochondrial fission promotes metabolic flexibility in TNBC cells, whereas mitochondrial fusion preserves bioenergetic stability. The coordinated balance between these opposing processes enables TNBC cells to maintain metabolic homeostasis amidst dynamic microenvironmental changes (27, 28). Under conditions of nutrient stress, Mechanistic target of mTORC1-mediated suppression of AMPK activity enhances lipid synthesis and mitochondrial energy production, allowing TNBC cells to sustain survival in nutrient-deprived environments (29).

Lipid metabolism is closely connected to mitochondrial function, particularly in FAO, which serves as a major energy source for TNBC cells (30). Fatty acids undergo CPT1 -mediated conversion to acylcarnitines for mitochondrial import, where sequential β-oxidation cycles generate acetyl-CoA and reducing equivalents (NADH/FADH_2_) that fuel the TCA cycle and oxidative phosphorylation. Beyond ATP production, this metabolic pathway critically regulates cellular metabolic plasticity through dynamic modulation of mitochondrial membrane potential (ΔΨm) and ROS homeostasis (27, 28). Jun Hyoung Park et al. (31), utilizing mitochondrial cell fusion models and multi-omics analyses, demonstrated that metastatic TNBC cells maintain high ATP levels via FAO, highlighting the pivotal role of mitochondrial FAO in TNBC. Furthermore, Stephen D. Williams et al. (32) found that reduced expression of Anxa6 in basal-like and mesenchymal-like TNBC cells accelerates FAO, thereby enhancing mitochondrial ATP production. Mokryun L. Baek et al. (33) further revealed that neoadjuvant chemotherapy (NACT) induces the expression of key lipid metabolism proteins and elevates mitochondrial oxidative phosphorylation, promoting lipid droplet accumulation in chemotherapy-resistant TNBC cells. Together, these studies underscore how TNBC cells enhance mitochondrial energy production by increasing fatty acid uptake and oxidation, enabling sustained proliferation and survival, particularly under stress conditions such as chemotherapy or nutrient deprivation. Beyond energy production, lipid metabolism intermediates—particularly fatty acids—directly impact mitochondrial membrane integrity and function, thus influencing intracellular energy metabolism. Fatty acids act as precursors for essential membrane components, including phospholipids and sphingolipids (34), which are vital for membrane synthesis and repair in rapidly proliferating tumor cells. Wang et al. (35) found that PdpaMn effectively targets breast cancer cells by inhibiting FASN, leading to apoptosis due to impaired mitochondrial membrane synthesis. Additionally, nicotinamide supplementation has been shown to enhance lipid metabolism while simultaneously promoting ROS induced mitochondrial dysfunction, ultimately leading to TNBC cell death (36).

In conclusion, lipid metabolism and mitochondrial bioenergetics are intricately interconnected, both playing essential roles in the metabolic regulation and progression of TNBC. Further exploration of their interplay may provide deeper insights into TNBC metabolism and facilitate the development of novel therapeutic strategies.

## 3 The role of lipid metabolism in metastasis and recurrence

### 3.1 Lipid metabolism in tumor cell migration and invasion

**Figure 2:**
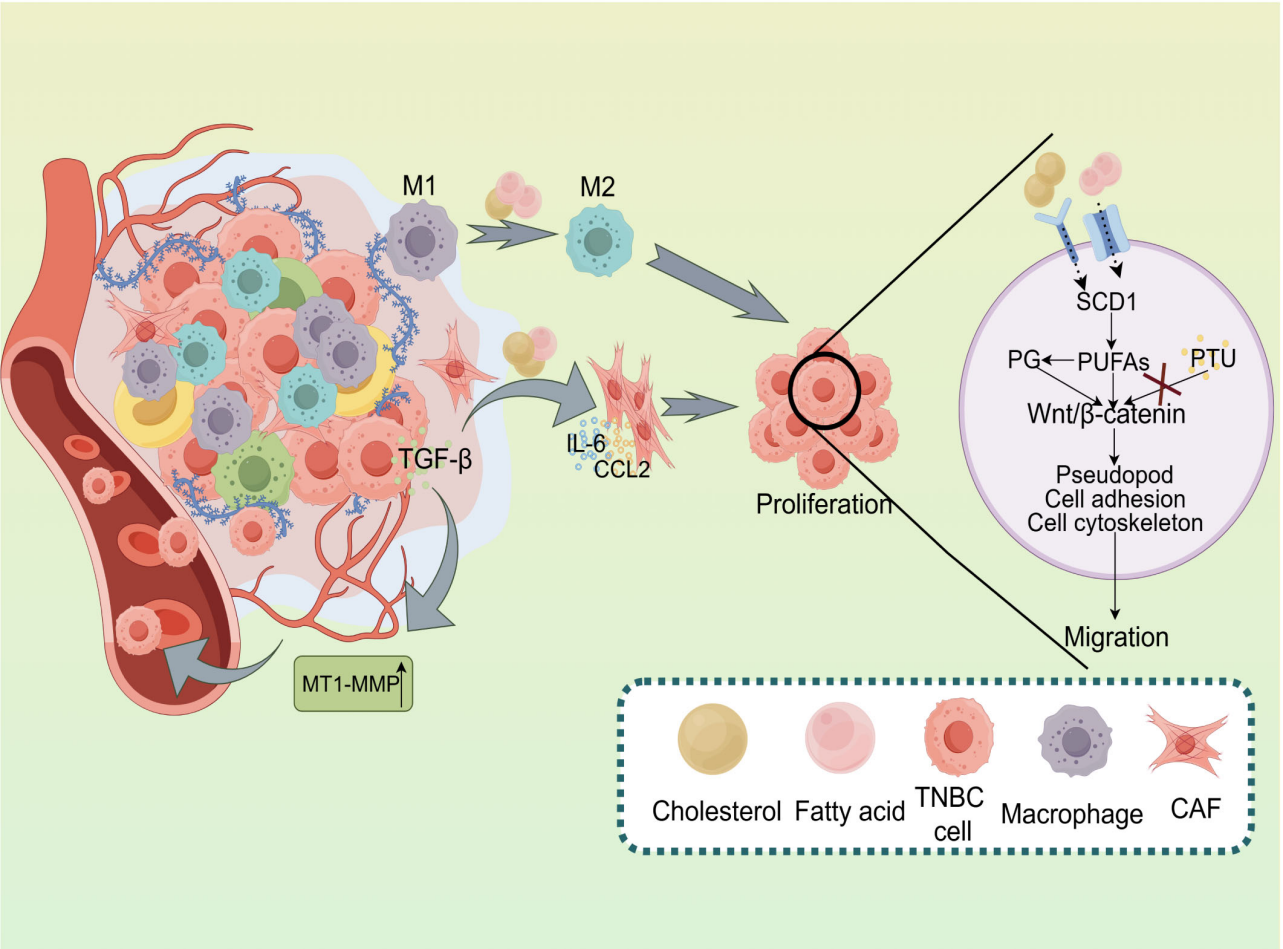
Impact of Lipid Metabolism on the Tumor Microenvironment and Metastasis in TNBC. Lipid metabolism plays a pivotal role in regulating the tumor microenvironment and facilitating tumor progression in TNBC. The activation of M1 and M2 macrophages, along with the secretion of key factors like TGF-β, IL-6, and CCL2, promotes TNBC cell proliferation. Additionally, lipid metabolism modulates Wnt/β-catenin signaling, influencing pseudopod formation, cell adhesion, and cytoskeletal dynamics, which collectively drive tumor cell migration. Enzymes such as SCD1 and PG regulatePUFAs, affecting various cellular processes. Conversely, the inhibition of lipid metabolism by PTU may decrease tumor cell migration. Moreover, MT1-MMP facilitates extracellular matrix remodeling, a critical process for tumor metastasis.

EMT is a crucial process through which tumor cells acquire enhanced migratory and invasive properties. In TNBC, EMT initiation is often linked to dysregulated lipid metabolism (**Figure 2**). During EMT, cells undergo a transition from an epithelial to a mesenchymal-like phenotype, increasing motility and invasiveness (37). Liu et al. (38) demonstrated that co-culture with adipocytes promotes TNBC cell proliferation and facilitates distant metastasis. Moreover, lipid metabolic byproducts—especially specific fatty acids—play a key role in EMT induction. For instance, alpha-linolenic acid (ALA) has been shown to inhibit TNBC cell migration by suppressing Twist1 expression and downregulating the EMT process (39).

Lipid metabolism supports TNBC cell migration by regulating cytoskeletal dynamics and producing bioactive lipid byproducts, particularly fatty acids, which further promote EMT (40). Alterations in fatty acid composition within membrane lipids can modulate membrane fluidity and membrane protein distribution, ultimately influencing cell morphology and motility. Studies have shown that the novel urea-based fatty acid derivative PTU inhibits MDA-MB-231 breast cancer cell migration and invasion by regulating Wnt5a secretion and cytoskeletal signaling pathways (41). Additionally, lipid metabolites such as sphingolipids and prostaglandins influence the expression of cell surface adhesion molecules, thereby affecting tumor cell adhesion to the extracellular matrix and adjacent cells. Research suggests that unsaturated fatty acids may alter breast cancer cell adhesion, potentially impacting metastatic spread and disease progression (42).

The role of lipid metabolism in TNBC migration and invasion is multifaceted. These mechanisms not only contribute to the metastatic phenotype of TNBC but also offer potential therapeutic targets. Targeting lipid metabolism pathways or their metabolic products presents a promising strategy for limiting tumor metastasis and improving clinical outcomes in TNBC patients.

### 3.2 Remodeling the tumor microenvironment

The tumor microenvironment is a highly dynamic and complex system composed of tumor cells, immune cells, fibroblasts, blood vessels, and the extracellular matrix. In TNBC, dysregulated lipid metabolism plays a pivotal role in shaping the tumor microenvironment by modifying cellular components and cytokine profiles, thereby promoting tumor angiogenesis and establishing a pre-metastatic niche that facilitates tumor progression and metastasis (**Figure 2**).

Fatty acids and lipid metabolic byproducts are key regulators of macrophage polarization, driving the transition from an anti-tumorigenic M1 phenotype to a pro-tumorigenic M2 phenotype (43). This shift modulates immune responses through cytokine and chemokine secretion, allowing tumor cells to evade immune surveillance and promoting immune escape. Studies have shown that fenofibrate (FF), a peroxisome PPAR-α agonist, enhances fatty acid catabolism and restores anti-tumor activity within the metabolically reprogrammed TNBC microenvironment (44). Tumor cells increase lipid uptake via the scavenger receptor CD36, which induces lipid peroxidation and dysfunction in CD8+ T cells, impairing antitumor immunity and promoting immune escape (45). Additionally, the lipid metabolite 4-hydroxynonenal (4-HNE) triggers apoptosis in T and B lymphocytes, further compromising antitumor immune responses (46). Lipid metabolism also suppresses antitumor immunity by driving the expansion of regulatory T cells (Tregs). Treg expansion is closely linked to dysregulated lipid metabolism, particularly in the TNBC tumor microenvironment, where fatty acid synthesis enhances Treg survival and differentiation. By releasing immunosuppressive cytokines, Tregs inhibit the activity of effector T cells and cytotoxic T cells, enabling TNBC cells to evade immune surveillance and exacerbating immune suppression (47). Moreover, TNBC cells utilize lipid metabolism-associated enzymes to modulate vascular morphology and function, thus enhancing metastatic capacity. Membrane-type 1 matrix metalloproteinase (MT1-MMP) activity is essential for vascular infiltration and distant metastasis in TNBC. Elevated MT1-MMP expression has been correlated with increased metastatic potential (48). Angiogenesis not only sustains tumor cell growth but also provides a direct route for tumor cells to enter the bloodstream, accelerating cancer dissemination. Cancer-associated fibroblasts (CAFs) contribute to tumor progression and are particularly resistant to TNBC therapy (49). Lipid metabolism also affects the function of CAFs, promoting their transition to a pro-tumorigenic phenotype (50). Increased fatty acid uptake and metabolism drive CAFs to secrete various factors that stimulate tumor cell proliferation, migration, and metastasis. Li et al. (51) found that reduced expression of retinoic acid receptor responder protein 2 (RARRES2) in brain-tropic TNBC cells enhances their survival in the unique brain microenvironment by regulating the PTEN-PI3K-SREBP1 signaling pathway. This metabolic adaptation is associated with increased glycerophospholipid levels and decreased triglyceride levels, facilitating tumor progression and metastatic colonization.

## 4 The interplay between lipid metabolism and cell death

**Figure 3:**
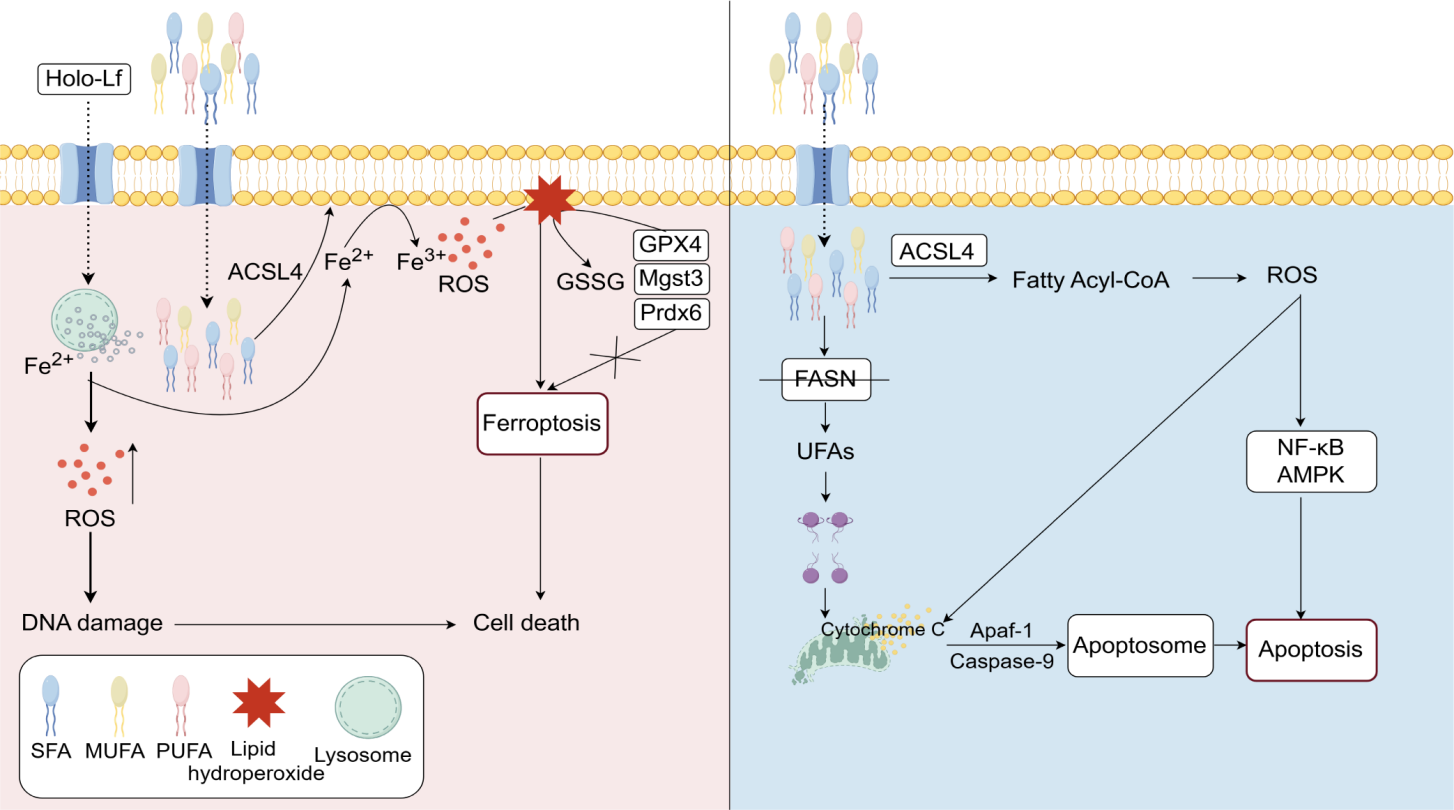
Lipid metabolism plays a crucial regulatory role in ferroptosis and apoptosis. Holo-Lf facilitates iron ion uptake, leading to the generation of ROS. The accumulation of ROS causes DNA damage, which triggers ferroptosis. The enzyme ACSL4 participates in lipid metabolism by regulating the balance of acyl-CoA, thereby promoting ferroptosis through lipid peroxidation. In contrast, FASN inhibition disrupts fatty acid synthesis, destabilizes mitochondrial membranes, induces cytochrome C release, and activates caspase-9, ultimately triggering apoptosis. The NF-κB and AMPK signaling pathways are involved in these processes, mediating the effects of ROS and cell death mechanisms.

Lipid metabolism plays a pivotal role in various cell death processes, including ferroptosis and apoptosis (**Figure 3**). Ferroptosis is an iron-dependent form of cell death characterized by the accumulation of lipid peroxides, particularly the peroxidation of polyunsaturated fatty acids (PUFAs) in cell membranes (52). In recent years, ferroptosis has attracted significant attention due to its role in tumor metabolic reprogramming and drug resistance. In TNBC, aberrant lipid metabolism can modulate ferroptosis signaling pathways, acting as a crucial determinant of tumor cell survival and death. Lipid metabolism is integral to ferroptosis, particularly through iron-catalyzed fatty acid peroxidation, which accelerates the ferroptotic process (8). Dysregulated lipid metabolism, especially excessive fatty acid accumulation, enhances lipid peroxidation, thereby activating ferroptosis-related signaling pathways. Research has shown that iron ions promote fatty acid peroxidation, leading to the accumulation of lipid peroxides and the activation of ferroptotic signaling cascades. The inhibition of glutathione peroxidase 4 (GPX4) is a key event in this process (53). Additionally, Mgst3 and Prdx6 encode glutathione-dependent peroxidases that detoxify lipid peroxides. Mutant p53 protects cells from ferroptosis by regulating Mgst3 and Prdx6 via the NRF pathway (54). These metabolic alterations disrupt the balance between cell survival and death, promoting ferroptotic cell demise. Regarding the mechanisms of drug resistance in TNBC, Zhang et al. (55) demonstrated that Holo-Lactoferrin (Holo-Lf) induces ferroptosis in TNBC cells. Holo-Lf binds to cell surface iron transporters, facilitating iron ion uptake and increasing intracellular iron levels. Elevated iron ions catalyze the peroxidation of PUFAs, producing excessive ROS. This leads to the accumulation of lipid peroxides, ultimately inducing ferroptosis, which sensitizes tumors to radiotherapy and improves its therapeutic efficacy. Wang et al. (56) found that Suppressor Of Cytokine Signaling 1 (SOCS1), a ferroptosis inhibitor, modulates ferroptosis by regulating GPX4 expression, thereby suppressing TNBC progression and chemoresistance.

In addition to ferroptosis, lipid metabolism also plays a critical role in apoptosis regulation. Apoptosis is a programmed cell death mechanism primarily mediated by caspase activation (57). In TNBC cells, lipid metabolism-associated products and enzymes influence cell fate by modulating multiple apoptotic pathways. Studies have shown that FASN inhibition enhances mitochondrial apoptosis, increasing TNBC cell death (58). Knockout of LPIN1 impairs phospholipid synthesis and alters membrane lipid composition, ultimately activating the inositol-requiring enzyme 1α pathway and promoting apoptosis in TNBC cells (59). Moreover, lipid peroxides have been implicated in apoptosis induction by promoting ROS production, which in turn activates apoptotic signaling pathways (60). He et al. (58) reported that co-administration of SBFI-26 and docetaxel significantly enhanced TNBC apoptosis by elevating intracellular ROS levels.

Dysregulated lipid metabolism influences tumor cell fate by altering fatty acid metabolism, promoting lipid peroxidation, and activating ferroptosis and apoptosis pathways. Consequently, targeting lipid metabolism and modulating cell death pathways may offer a promising therapeutic strategy for TNBC treatment.

## 5 Clinical approaches to targeting lipid metabolism in TNBC

Lipid metabolism enzymes and their metabolic byproducts play critical roles in the progression of TNBC, including tumor proliferation, metastasis, and immune evasion. Targeting these molecules represents a promising therapeutic strategy (3). The overexpression of key enzymes such as Acyl-CoA Synthetase Long Chain Family Member 4 (ACSL4), FASN, and HMG-CoA in TNBC cells is strongly associated with tumor proliferation and metastasis. Research indicates that ACSL4 upregulation in TNBC is also closely linked to tumor metastasis. ACSL4, a critical enzyme in polyunsaturated fatty acid (PUFA) biosynthesis, catalyzes the conversion of PUFAs to acyl-CoA, facilitating fatty acid activation. This process enhances cell membrane fluidity, promoting tumor metastasis (61). However, as the primary substrate for lipid peroxidation, increased PUFA levels heighten TNBC cell sensitivity to ferroptosis, inducing cell death (62). Furthermore, ACSL4 catalyzes arachidonic acid to produce the lipid metabolism intermediate 5-hydroxyeicosatetraenoic acid (5-HETE), whose accumulation triggers cell membrane lipid peroxidation, leading to ferroptosis and suppressing tumor cell survival (63). This dual role suggests that ACSL4’s function in TNBC may depend on its regulatory mechanisms within different metabolic contexts. Consequently, therapeutic strategies targeting ACSL4 must be tailored to the specific tumor microenvironment. Given the heterogeneity in lipid metabolism pathways across TNBC subtypes, lipid metabolism-targeted therapies also vary. For instance, the MPS1 subtype, characterized by upregulated lipid metabolism, can be targeted with FASN inhibitors to block fatty acid synthesis, reducing tumor cell energy supply and membrane lipid production, thereby inhibiting tumor growth. Inhibitors of enzymes such as ACACA and HMGCR can also disrupt cholesterol and steroid biosynthesis, impairing tumor cell proliferation and survival (3). These findings offer novel insights and strategies for precision therapy in TNBC.

Inhibiting the activity of these enzymes effectively blocks lipid synthesis pathways, depleting tumor cell energy sources and suppressing growth and proliferation. Currently, inhibitors targeting these key enzymes are under development (**Table 1**). For example, lipid nanoparticle-based gene therapy can suppress tumor-associated adipocytes and remodel the immunosuppressive tumor microenvironment in TNBC (64). In studies using brain-derived TNBC cell lines, the combination of SN-38 and TVB-2640 demonstrated targeted inhibition of brain metastasis (65). TVB-2640 demonstrated safety and efficacy in clinical trials for HER2-positive metastatic breast cancer (NCT03179904) and non-alcoholic steatohepatitis (NCT03938346, NCT02948569). Similarly, NDI-010976, an acetyl-CoA carboxylase (ACC) inhibitor, exhibited potential in treating obesity in clinical trial NCT02876796. HMGCR regulates intracellular cholesterol levels, and statins inhibit sterol biosynthesis through the mevalonate pathway, thereby lowering lipid levels. Furthermore, clinical trial NCT03358017 revealed that combining zoledronate with statins enhances antitumor efficacy in TNBC patients undergoing NACT.

**Table 1:** Clinical development status of lipid metabolism-targeting therapies in triple-negative breast cancer.

Target	Agent/Therapy	Mechanism of action	Development stage	Clinical trial identifier	Key findings
FASN	TVB-2640	Fatty acid synthase inhibitor	Phase II	NCT03179904	Demonstrated activity in HER2+ metastatic BC
ACSL4	Under development	Modulates PUFA metabolism	Preclinical	–	Context-dependent pro-metastatic/ferroptotic effects
HMGCR	Atorvastatin + Zoledronate	HMG-CoA reductase inhibitor combination	Phase II	NCT03358017	Showed antitumor activity in neoadjuvant setting
ACC	NDI-010976	Acetyl-CoA carboxylase inhibitor	Phase I	NCT02876796	Potential metabolic effects observed
Sphingolipid metabolism	Investigational	Modulates membrane signaling	Preclinical	–	Correlation with improved DFS observed

Targeting lipid metabolic byproducts also represents a viable therapeutic approach. Prostaglandins and sphingolipids are lipid metabolites that play pivotal roles in cancer cell signaling, proliferation, and survival. Inhibiting these metabolic products can disrupt oncogenic signaling pathways and impede tumor progression. FAO has emerged as a potential therapeutic target for MYC-overexpressing TNBC, as its inhibition reduces energy metabolism and impairs tumor growth (66). Furthermore, emerging research suggests that sphingolipid-targeting therapies could be beneficial, as sphingolipids significantly influence tumor cell membrane dynamics and signal transduction. Multivariate survival analysis has shown that elevated sphingomyelin levels are associated with improved disease-free survival in TNBC patients, highlighting sphingomyelin as a potential therapeutic target (67). In addition, Chen et al. found that CALU is highly expressed in TNBC and is involved in tumor metastasis and microenvironment regulation. Research on CALU as a potential target helps further understand how lipid metabolism promotes the progression of TNBC (68).

## 6 Analytical approaches for investigating lipid metabolism in TNBC

### 6.1 Application of multi-omics technologies

Transcriptomic analysis, enabled by high-throughput sequencing technologies, facilitates the identification of gene expression changes associated with lipid metabolism. In TNBC cells, alterations in lipid metabolism often coincide with the upregulation or downregulation of genes involved in fatty acid synthesis, β-oxidation, and cholesterol metabolism. Transcriptomic studies systematically identify key lipid metabolism-related genes, providing valuable insights into their roles in tumor metabolic reprogramming. Gong et al. (3) used metabolic pathway-based TNBC subtyping and polymorphic database analysis to identify potential therapeutic targets for TNBC. Bassiouni et al. (69) applied spatial transcriptomics to investigate TNBC tumor architecture, shedding light on racial disparities in TNBC. Chen et al. (70) integrated machine learning with transcriptomic analysis to identify lipid metabolism-related genes in breast cancer, offering novel insights into therapeutic strategies and molecular mechanisms. These findings underscore the significance of transcriptomics in characterizing the metabolic heterogeneity of TNBC, identifying prognostic and predictive biomarkers, and informing the development of targeted therapies and personalized treatment approaches.

Lipidomics is a powerful tool for studying lipid composition, distribution, and metabolism, enabling a comprehensive analysis of intracellular lipid profiles and metabolic alterations. Techniques such as liquid chromatography-mass spectrometry (LC-MS) allow for the quantitative assessment of fatty acids, phospholipids, sphingolipids, and triglycerides in TNBC cells. Recent studies have demonstrated that LC-MS/MS-based lipidomic analysis enables precise and highly sensitive differentiation of TNBC subtypes, while also identifying dysregulated choline metabolism, sphingolipid signaling, and glycerophospholipid metabolism (71). Additionally, single-cell mass spectrometry using CyESI-MS has been employed to analyze unsaturated lipid profiles, revealing heterogeneity in polyunsaturated lipid composition in TNBC cells (72). Lipidomic technologies provide valuable insights into TNBC diagnosis, metabolic heterogeneity, and the identification of lipid metabolism-related therapeutic targets, including those associated with chemotherapy resistance.

Proteomics plays a crucial role in identifying key enzymes and regulatory proteins involved in lipid metabolism. Quantitative proteomic analysis offers insights into the expression patterns of lipid metabolism-associated enzymes and their impact on tumor proliferation, migration, and therapy resistance. Proteomic studies have been employed to investigate FASN expression under different conditions, elucidating its role in lipid metabolism (12). Additionally, proteomics has been used to examine MT1-MMP expression in TNBC cells and its involvement in tumor metastasis (48).

By integrating transcriptomics, lipidomics, and proteomics, researchers can construct comprehensive regulatory networks of lipid metabolism. The advancements in multi-omics technologies have revolutionized the analysis of tumor heterogeneity and the immune microenvironment in TNBC (73). Yang et al. (74) employed multi-omics analyses to highlight the potential of targeting the SCD1-TRIM28-PD-L1 axis to overcome chemotherapy resistance in TNBC. Multi-omics approaches provide a systems-level understanding of lipid metabolism in TNBC, encompassing alterations in lipid synthesis, oxidation, and storage, while elucidating how these changes drive tumor growth, metastasis, and therapy resistance.

### 6.2 Imaging techniques for monitoring lipid metabolism

Imaging technologies play a pivotal role in lipid metabolism research by enabling the real-time monitoring of dynamic metabolic changes in cancer cells (75). These techniques facilitate the spatial tracking of lipid metabolism by mapping variations in lipid metabolic byproducts, providing valuable insights into the metabolic activity of tumor cells. In addition to identifying lipid metabolism hotspots within the tumor microenvironment, imaging techniques also serve as essential clinical tools for tracking tumor progression and evaluating therapeutic efficacy (76). Anthony A. Fung et al. (77) employed 3D spatial and chemometric analysis to uncover distinct lipid metabolic profiles in TNBC, highlighting potential therapeutic targets and establishing a powerful subcellular imaging platform for metabolism and cancer research. The integration of Raman resonance spectroscopy (RRS) and multiphoton fluorescence (MPF) microscopy has been shown to enhance the accuracy of early TNBC metabolic detection, thereby improving diagnostic precision (78). Furthermore, dual-output stimulated Raman scattering (DO-SRS) imaging enables subcellular visualization and quantification of metabolic activity across different TNBC subtypes, contributing to advancements in early detection and treatment optimization (79).

Collectively, these studies highlight the utility of imaging techniques in characterizing lipidomic alterations, metabolic reprogramming, and the identification of novel therapeutic targets in TNBC.

### 6.3 Investigating lipid metabolism in TNBC using cell and animal models

*In vitro* cell culture models provide a platform for simulating the lipid metabolism characteristics of TNBC cells and examining how metabolic alterations affect tumor cell behavior. Studies utilizing FAO inhibitors have demonstrated that FAO suppression reduces TNBC cell proliferation, emphasizing the crucial role of lipid metabolism in tumor cell survival (16). Furthermore, researchers have observed that treatment with ROS inducers, such as SBFI-26 and docetaxel, significantly increases intracellular ROS levels in TNBC cells, thereby promoting apoptosis (58). These *in vitro* findings establish a critical link between lipid metabolism and the regulation of cell death.

Animal models are indispensable tools for investigating the role of lipid metabolism in tumor initiation, progression, and response to therapy. *In vivo* studies allow for the assessment of therapeutic interventions targeting lipid metabolism in TNBC. In a NACT mouse model, chemotherapy was found to induce the upregulation of lipid metabolism proteins and enhance mitochondrial oxidative phosphorylation, leading to increased lipid droplet accumulation in surviving TNBC cells (33). Additionally, in mouse models, CAFs contribute to tumor progression by uptaking and metabolizing fatty acids to produce secretory factors that stimulate tumor cell proliferation, migration, and metastasis (51). These models provide critical insights into the role of lipid metabolism in tumor progression and serve as a foundation for evaluating novel therapeutic strategies.

## 7 Conclusion and future directions

Future research should focus on further elucidating the role of lipid metabolism in different TNBC subtypes, identifying novel therapeutic targets, and optimizing existing treatment strategies. The integration of multi-omics analyses with advanced imaging technologies will provide deeper insights into the dynamic alterations in lipid metabolism and its intricate crosstalk with the tumor microenvironment. Single-cell technologies are particularly suited for dissecting lipid metabolism heterogeneity among TNBC cells and identifying critical metabolic pathways across different subtypes. Single-cell lipidomics enables the analysis of variations in fatty acid synthesis, cholesterol metabolism, and phospholipid remodeling, uncovering metabolic features of drug-resistant cell subpopulations. Spatial metabolomics provides insights into the spatial distribution of lipid metabolites within the tumor microenvironment, facilitating the exploration of metabolic heterogeneity among TNBC cell subpopulations and interactions between lipid metabolism and microenvironmental components, such as immune cells and fibroblasts. Notably, it reveals spatial co-localization patterns between lipid metabolites and tumor-associated macrophages or tumor-infiltrating lymphocytes. When integrated with spatial transcriptomics, this approach further elucidates the relationship between localized gene expression and lipid metabolism, offering a theoretical foundation for developing precision therapies targeting lipid metabolism. Additionally, the integration of multi-omics data allows for the construction of dynamic network models of TNBC lipid metabolism, advancing the development of targeted therapeutic strategies. These efforts hold promise for improving the prognosis of TNBC patients and offering novel therapeutic strategies, particularly for those resistant to conventional therapies.

## References

[1] Borri F Granaglia A. Pathology of triple negative breast cancer. Semin Cancer Biol. (2021) 72:136–45. doi: 10.1016/j.semcancer.2020.06.005 32544511

[2] Lehmann BD Jovanović B Chen X Estrada MV Johnson KN Shyr Y. Refinement of triple-negative breast cancer molecular subtypes: implications for neoadjuvant chemotherapy selection. PloS One. (2016) 11:e0157368. doi: 10.1371/journal.pone.0157368 27310713 PMC4911051

[3] Gong Y Ji P Yang YS Xie S Yu TJ Xiao Y. Metabolic-pathway-based subtyping of triple-negative breast cancer reveals potential therapeutic targets. Cell Metab. (2021) 33:51–64.e9. doi: 10.1016/j.cmet.2020.10.012 33181091

[4] Koundouros N Poulogiannis G. Reprogramming of fatty acid metabolism in cancer. Br J Cancer. (2020) 122:4–22. doi: 10.1038/s41416-019-0650-z 31819192 PMC6964678

[5] Bian X Liu R Meng Y Xing D Xu D Lu Z. Lipid metabolism and cancer. J Exp Med. (2020) 218:e20201606. doi: 10.1084/jem.20201606 PMC7754673 33601415

[6] Fu W Sun A Dai H. Lipid metabolism involved in progression and drug resistance of breast cancer. Genes Dis. (2024) 12(4):101376. doi: 10.1016/j.gendis.2024.101376 40256431 PMC12008617

[7] Corn KC Windham MA Rafat M. Lipids in the tumor microenvironment: From cancer progression to treatment. Prog Lipid Res. (2020) 80:101055. doi: 10.1016/j.plipres.2020.101055 32791170 PMC7674189

[8] Wang Y Sun Y Wang F Wang H Hu J. Ferroptosis induction via targeting metabolic alterations in triple-negative breast cancer. BioMed Pharmacother. (2023) 169:115866. doi: 10.1016/j.biopha.2023.115866 37951026

[9] Guan X Meng X Zhu K Kai J Liu Y Ma Q. MYSM1 induces apoptosis and sensitizes TNBC cells to cisplatin via RSK3-phospho-BAD pathway. Cell Death Discov. (2022) 8:84. doi: 10.1038/s41420-022-00881-1 35217648 PMC8881619

[10] Wang Z Jiang Q Dong C. Metabolic reprogramming in triple-negative breast cancer. Cancer Biol Med. (2020) 17:44–59. doi: 10.20892/j.issn.2095-3941.2019.0210 32296576 PMC7142847

[11] Buckley D Duke G Heuer TS O’Farrell M Wagman AS McCulloch W. Fatty acid synthase - Modern tumor cell biology insights into a classical oncology target. Pharmacol Ther. (2017) 177::23–31. doi: 10.1016/j.pharmthera.2017.02.021 28202364

[12] Park JH Han HS Lim SD Kim WY Park KS Yoo YB. Fatty acid synthetase expression in triple-negative breast cancer. J Pathol Transl Med. (2022) 56:73–80. doi: 10.4132/jptm.2021.10.27 35051326 PMC8935000

[13] Gonzalez-Salinas F Rojo R Martinez-Amador C Herrera-Gamboa J Trevino V. Transcriptomic and cellular analyses of CRISPR/Cas9-mediated edition of FASN show inhibition of aggressive characteristics in breast cancer cells. Biochem Biophys Res Commun. (2020) 529:321–7. doi: 10.1016/j.bbrc.2020.05.172 32703430

[14] Ruidas B Choudhury N Chaudhury SS Sur TK Bhowmick S Saha A. Precision targeting of fat metabolism in triple negative breast cancer with a biotinylated copolymer. J Mater Chem B. (2025) 13:1363–71. doi: 10.1039/d4tb02142h 39661021

[15] Ricoult SJH Yecies JL Ben-Sahra I Manning BD. Oncogenic PI3K and K-Ras stimulate *de novo* lipid synthesis through mTORC1 and SREBP. Oncogene. (2016) 35:1250–60. doi: 10.1038/onc.2015.179 PMC4666838 26028026

[16] Blücher C Iberl S Schwagarus N Müller S Liebisch G Höring M. Secreted factors from adipose tissue reprogram tumor lipid metabolism and induce motility by modulating PPARα/ANGPTL4 and FAK. Mol Cancer Res. (2020) 18:1849–62. doi: 10.1158/1541-7786.MCR-19-1223 32859692

[17] Woodcock CSC Huang Y Woodcock SR Salvatore SR Singh B Golin-Bisello F. Nitro-fatty acid inhibition of triple-negative breast cancer cell viability, migration, invasion, and tumor growth. J Biol Chem. (2018) 293:1120–37. doi: 10.1074/jbc.M117.814368 PMC5787792 29158255

[18] Friesen JA Rodwell VW. The 3-hydroxy-3-methylglutaryl coenzyme-A (HMG-CoA) reductases. Genome Biol. (2004) 5:248. doi: 10.1186/gb-2004-5-11-248 15535874 PMC545772

[19] Huang B Song BL Xu C. Cholesterol metabolism in cancer: mechanisms and therapeutic opportunities. Nat Metab. (2020) 2:132–41. doi: 10.1038/s42255-020-0174-0 32694690

[20] Goupille C Ouldamer L Pinault M Guimares C Arbion F Jourdan ML. Identification of a positive association between mammary adipose cholesterol content and indicators of breast cancer aggressiveness in a french population. J Nutr. (2021) 151:1119–27. doi: 10.1093/jn/nxaa432 33831951

[21] Chen M Zhao Y Yang X Zhao Y Liu Q Liu Y. NSDHL promotes triple-negative breast cancer metastasis through the TGFβ signaling pathway and cholesterol biosynthesis. Breast Cancer Res Treat. (2021) 187:349–62. doi: 10.1007/s10549-021-06213-8 33864166

[22] Gao W Guo X Sun L Gai J Cao Y Zhang S. PKMYT1 knockdown inhibits cholesterol biosynthesis and promotes the drug sensitivity of triple-negative breast cancer cells to atorvastatin. PeerJ. (2024) 12:e17749. doi: 10.7717/peerj.17749 39011373 PMC11249011

[23] O’Neill K. SAT-145 cholesterol uptake as a critical vulnerability in triple negative breast cancer. J Endocrine Soc. (2020) 4:SAT–145. doi: 10.1210/jendso/bvaa046.1368

[24] Ghanbari F Fortier AM Park M Philip A. Cholesterol-induced metabolic reprogramming in breast cancer cells is mediated via the ERRα Pathway. Cancers (Basel). (2021) 13:2605. doi: 10.3390/cancers13112605 34073320 PMC8198778

[25] Etella A Scully T Jordan S Kang C Jeffrey MG Kase NG. OR16-05 25-hydroxycholesterol mediates the effects of dyslipidemia on triple negative breast cancer metastasis. J Endocrine Soc. (2023) 7:bvad114.2197. doi: 10.1210/jendso/bvad114.2197

[26] Cai D Wang J Gao B Li J Wu F Zou JX. RORγ is a targetable master regulator of cholesterol biosynthesis in a cancer subtype. Nat Commun. (2019) 10:4621. doi: 10.1038/s41467-019-12529-3 31604910 PMC6789042

[27] Ruidas B. Mitochondrial lipid metabolism in metastatic breast cancer. Mitochondrial Commun. (2024) 2:58–66. doi: 10.1016/j.mitoco.2024.07.001

[28] Di Gregorio J Petricca S Iorio R Toniato E Flati V. Mitochondrial and metabolic alterations in cancer cells. Eur J Cell Biol. (2022) 101:151225. doi: 10.1016/j.ejcb.2022.151225 35453093

[29] Ling NXY Kaczmarek A Hoque A Davie E Ngoei KRW Morrison KR. mTORC1 directly inhibits AMPK to promote cell proliferation under nutrient stress. Nat Metab. (2020) 2:41–9. doi: 10.1038/s42255-019-0157-1 PMC6986917 31993556

[30] Guerra IMS Ferreira HB Melo T Rocha H Moreira S Diogo L. Mitochondrial fatty acid β-oxidation disorders: from disease to lipidomic studies-A critical review. Int J Mol Sci. (2022) 23:13933. doi: 10.3390/ijms232213933 36430419 PMC9696092

[31] Park JH Vithayathil S Kumar S Sung PL Dobrolecki LE Putluri V. Fatty acid oxidation-driven src links mitochondrial energy reprogramming and oncogenic properties in triple-negative breast cancer. Cell Rep. (2016) 14:2154–65. doi: 10.1016/j.celrep.2016.02.004 PMC4809061 26923594

[32] Williams SD Sakwe AM. Reduced expression of annexin A6 induces metabolic reprogramming that favors rapid fatty acid oxidation in triple-negative breast cancer cells. Cancers (Basel). (2022) 14:1108. doi: 10.3390/cancers14051108 35267416 PMC8909273

[33] Baek ML Lee J Pendleton KE Berner MJ Goff EB Tan L. Mitochondrial structure and function adaptation in residual triple negative breast cancer cells surviving chemotherapy treatment. Oncogene. (2023) 42:1117–31. doi: 10.1038/s41388-023-02596-8 PMC10069007 36813854

[34] Jin L Kamat NP Jena S Szostak JW. Fatty acid/phospholipid blended membranes: A potential intermediate state in protocellular evolution. Small. (2018) 14:e1704077. doi: 10.1002/smll.201704077 29479815 PMC6278924

[35] Wang Q Du X Zhou B Li J Lu W Chen Q. Mitochondrial dysfunction is responsible for fatty acid synthase inhibition-induced apoptosis in breast cancer cells by PdpaMn. BioMed Pharmacother. (2017) 96:396–403. doi: 10.1016/j.biopha.2017.10.008 29031197

[36] Jung M Lee KM Im Y Seok SH Chung H Kim DY. Nicotinamide (niacin) supplement increases lipid metabolism and ROS-induced energy disruption in triple-negative breast cancer: potential for drug repositioning as an anti-tumor agent. Mol Oncol. (2022) 16:1795–815. doi: 10.1002/1878-0261.13209 PMC9067146 35278276

[37] Datta A Deng S Gopal V Yap KC Halim CE Lye ML. Cytoskeletal dynamics in epithelial-mesenchymal transition: insights into therapeutic targets for cancer metastasis. Cancers (Basel). (2021) 13:1882. doi: 10.3390/cancers13081882 33919917 PMC8070945

[38] Liu M Zhang Q. Polydatin ameliorates low-density lipoprotein cholesterol and lipid metabolism by downregulating proprotein convertase subtilisin/kexin type 9 (PCSK9) in triple-negative breast cancer with hyperlipidemia. Am J Cancer Res. (2024) 14:52–72. doi: 10.62347/BRNK8140 38323270 PMC10839302

[39] Wang SC Sun HL Hsu YH Liu SH Lii CK Tsai CH. α-Linolenic acid inhibits the migration of human triple-negative breast cancer cells by attenuating Twist1 expression and suppressing Twist1-mediated epithelial-mesenchymal transition. Biochem Pharmacol. (2020) 180:114152. doi: 10.1016/j.bcp.2020.114152 32679125

[40] Kwong SC Jamil AHA Rhodes A Taib NA Chung I. Metabolic role of fatty acid binding protein 7 in mediating triple-negative breast cancer cell death via PPAR-α signaling. J Lipid Res. (2019) 60:1807–17. doi: 10.1194/jlr.M092379 PMC6824484 31484694

[41] Al-Zubaidi Y Chen Y Khalilur Rahman M Umashankar B Choucair H Bourget K. PTU, a novel ureido-fatty acid, inhibits MDA-MB-231 cell invasion and dissemination by modulating Wnt5a secretion and cytoskeletal signaling. Biochem Pharmacol. (2021) 192:114726. doi: 10.1016/j.bcp.2021.114726 34389322

[42] Johanning GL. Modulation of breast cancer cell adhesion by unsaturated fatty acids. Nutrition. (1996) 12:810–6. doi: 10.1016/s0899-9007(96)00244-4 8974109

[43] Morgan PK Huynh K Pernes G Miotto PM Mellett NA Giles C. Macrophage polarization state affects lipid composition and the channeling of exogenous fatty acids into endogenous lipid pools. J Biol Chem. (2021) 297:101341. doi: 10.1016/j.jbc.2021.101341 34695418 PMC8604758

[44] Gu Y Niu X Yin L Wang Y Yang Y Yang X. Enhancing fatty acid catabolism of macrophages within aberrant breast cancer tumor microenvironment can re-establish antitumor function. Front Cell Dev Biol. (2021) 9:665869. doi: 10.3389/fcell.2021.665869 33937269 PMC8081981

[45] Xu S Chaudhary O Rodríguez-Morales P Sun X Chen D Zappasodi R. Uptake of oxidized lipids by the scavenger receptor CD36 promotes lipid peroxidation and dysfunction in CD8+ T cells in tumors. Immunity. (2021) 54:1561–1577.e7. doi: 10.1016/j.immuni.2021.05.003 34102100 PMC9273026

[46] Kalinich JF Ramakrishnan R McClain DE Ramakrishnan N. 4-Hydroxynonenal, an end-product of lipid peroxidation, induces apoptosis in human leukemic T- and B-cell lines. Free Radic Res. (2000) 33:349–58. doi: 10.1080/10715760000300891 11022844

[47] Malla RR Vasudevaraju P Vempati RK Rakshmitha M Merchant N Nagaraju GP. Regulatory T cells: Their role in triple-negative breast cancer progression and metastasis. Cancer. (2022) 128:1171–83. doi: 10.1002/cncr.34084 34990009

[48] Perentes JY Kirkpatrick ND Nagano S Smith EY Shaver CM Sgroi D. Cancer cell-associated MT1-MMP promotes blood vessel invasion and distant metastasis in triple-negative mammary tumors. Cancer Res. (2011) 71:4527–38. doi: 10.1158/0008-5472.CAN-10-4376 21571860

[49] Fiori ME Di Franco S Villanova L Bianca P Stassi G De Maria R. Cancer-associated fibroblasts as abettors of tumor progression at the crossroads of EMT and therapy resistance. Mol Cancer. (2019) 18:70. doi: 10.1186/s12943-019-0994-2 30927908 PMC6441236

[50] Li Z Sun C Qin Z. Metabolic reprogramming of cancer-associated fibroblasts and its effect on cancer cell reprogramming. Theranostics. (2021) 11:8322–36. doi: 10.7150/thno.62378 PMC8343997 34373744

[51] Li YQ Sun FZ Li CX Mo HN Zhou YT Lv D. RARRES2 regulates lipid metabolic reprogramming to mediate the development of brain metastasis in triple negative breast cancer. Mil Med Res. (2023) 10:34. doi: 10.1186/s40779-023-00470-y 37491281 PMC10369725

[52] Bayır H Anthonymuthu TS Tyurina YY Patel SJ Amoscato AA Lamade AM. Achieving life through death: redox biology of lipid peroxidation in ferroptosis. Cell Chem Biol. (2020) 27:387–408. doi: 10.1016/j.chembiol.2020.03.014 32275865 PMC7218794

[53] Zhang J Zhang S Liu M Yang Z Huang R. Research progress on ferroptosis and nanotechnology-based treatment in triple-negative breast cancer. Breast Cancer (Dove Med Press). (2024) 16:347–58. doi: 10.2147/bctt.s475199 PMC11268712 39050766

[54] Dibra D Xiong S Moyer SM El-Naggar AK Qi Y Su X. Mutant p53 protects triple-negative breast adenocarcinomas from ferroptosis *in vivo* . Sci Adv. (2024) 11(7):3167–82. doi: 10.1126/sciadv.adk1835 PMC10866549 38354236

[55] Zhang Z Lu M Chen C Tong X Li Y Yang K. Holo-lactoferrin: the link between ferroptosis and radiotherapy in triple-negative breast cancer. Theranostics (2021) 11(7):3167–82. doi: 10.7150/thno.52028 PMC7847686 33537080

[56] Wang Y Pang X Liu Y Mu G Wang Q. SOCS1 acts as a ferroptosis driver to inhibit the progression and chemotherapy resistance of triple-negative breast cancer. Carcinogenesis. (2023) 44:708–15. doi: 10.1093/carcin/bgad060 37665951

[57] McComb S Chan PK Guinot A Hartmannsdottir H Jenni S Dobay MP. Efficient apoptosis requires feedback amplification of upstream apoptotic signals by effector caspase-3 or -7. Sci Adv. (2019) 5:eaau9433. doi: 10.1126/sciadv.aau9433 31392262 PMC6669006

[58] Schroeder B Vander Steen T Espinoza I Venkatapoorna CMK Hu Z Silva FM. Fatty acid synthase (FASN) regulates the mitochondrial priming of cancer cells. Cell Death Dis. (2021) 12:977. doi: 10.1038/s41419-021-04262-x 34675185 PMC8531299

[59] He J Zhang F Tay LWR Boroda S Nian W Levental KR. Lipin-1 regulation of phospholipid synthesis maintains endoplasmic reticulum homeostasis and is critical for triple-negative breast cancer cell survival. FASEB J. (2017) 31:2893–904. doi: 10.1096/fj.201601353R PMC6137500 28347999

[60] Su LJ Zhang JH Gomez H Murugan R Hong X Xu D. Reactive oxygen species-induced lipid peroxidation in apoptosis, autophagy, and ferroptosis. Oxid Med Cell Longev. (2019) 2019:5080843. doi: 10.1155/2019/5080843 31737171 PMC6815535

[61] Qiu Y Wang X Sun Y Jin T Tang R Zhou X. ACSL4-mediated membrane phospholipid remodeling induces integrin β1 activation to facilitate triple-negative breast cancer metastasis. Cancer Res. (2024) 84:1856–71. doi: 10.1158/0008-5472.CAN-23-2491 PMC11148537 38471082

[62] Wang Y Hu M Cao J Wang F Han JR Wu TW. ACSL4 and polyunsaturated lipids support metastatic extravasation and colonization. Cell. (2025) 188:412–429.e27. doi: 10.1016/j.cell.2024.10.047 39591965

[63] Yuan H Li X Zhang X Kang R Tang D. Identification of ACSL4 as a biomarker and contributor of ferroptosis. Biochem Biophys Res Commun. (2016) 478:1338–43. doi: 10.1016/j.bbrc.2016.08.124 27565726

[64] Liu Y Tiruthani K Wang M Zhou X Qiu N Xiong Y. Tumor-targeted gene therapy with lipid nanoparticles inhibits tumor-associated adipocytes and remodels the immunosuppressive tumor microenvironment in triple-negative breast cancer. Nanoscale Horiz. (2021) 6:319–29. doi: 10.1039/d0nh00588f PMC8638658 33587080

[65] Serhan HA Bao L Cheng X Qin Z Liu CJ Heth JA. Targeting fatty acid synthase in preclinical models of TNBC brain metastases synergizes with SN-38 and impairs invasion. NPJ Breast Cancer. (2024) 10:43. doi: 10.1038/s41523-024-00656-0 38858374 PMC11164988

[66] Camarda R Zhou AY Kohnz RA Balakrishnan S Mahieu C Anderton B. Inhibition of fatty acid oxidation as a therapy for MYC-overexpressing triple-negative breast cancer. Nat Med. (2016) 22:427–32. doi: 10.1038/nm.4055 PMC4892846 26950360

[67] Purwaha P Gu F Piyarathna DWB Rajendiran T Ravindran A Omilian AR. Unbiased lipidomic profiling of triple-negative breast cancer tissues reveals the association of sphingomyelin levels with patient disease-free survival. Metabolites. (2018) 8:41. doi: 10.3390/metabo8030041 30011843 PMC6161031

[68] Chen SL Hu D Chen TZ Shen SY Zhao CF Wang C. Pan-cancer screening and validation of CALU’s role in EMT regulation and tumor microenvironment in triple-negative breast cancer. J Inflammation Res. (2024) 17:6743–64. doi: 10.2147/jir.s477846 PMC11439346 39345892

[69] Bassiouni R Idowu MO Gibbs LD Robila V Grizzard PJ Webb MG. Spatial transcriptomic analysis of a diverse patient cohort reveals a conserved architecture in triple-negative breast cancer. Cancer Res. (2023) 83:34–48. doi: 10.1158/0008-5472.can-22-2682 36283023 PMC9812886

[70] Chen X Yi J Xie L Liu T Liu B Yan M. Integration of transcriptomics and machine learning for insights into breast cancer: exploring lipid metabolism and immune interactions. Front Immunol. (2024) 15:1470167. doi: 10.3389/fimmu.2024.1470167 39524444 PMC11543460

[71] Eghlimi R Shi X Hrovat J Xi B Gu H. Triple negative breast cancer detection using LC-MS/MS lipidomic profiling. J Proteome Res. (2020) 19:2367–78. doi: 10.1021/acs.jproteome.0c00038 32397718

[72] Yang J Cheng R Pan X Pan S Du M Yao H. Single-cell unsaturated lipid profiling for studying chemoresistance heterogeneity of triple-negative breast cancer cells. Anal Chem. (2024) 10.1021/acs.analchem.3c04. doi: 10.1021/acs.analchem.3c04887 38334074

[73] Chen SL Fei YR Cai XX Wang C Tong SY Zhang ZZ. Exploring the role of metabolic pathways in TNBC immunotherapy: insights from single-cell and spatial transcriptomics. Front Endocrinol (Lausanne). (2024) 15:1528248. doi: 10.3389/fendo.2024.1528248 39850483 PMC11754047

[74] Yang S Tang J Zhang J. Overcoming chemoresistance in triple-negative breast cancer (TNBC): Targeting PD-L1 stability and ferroptosis through integrated lipid metabolism and immune modulation. JCO. (2024) 42:e15149-e15149 doi: 10.1200/jco.2024.42.16_suppl.e15149

[75] Hou J Reid NE Tromberg BJ Potma EO. Kinetic analysis of lipid metabolism in breast cancer cells via nonlinear optical microscopy. Biophys J. (2020) 119:258–64. doi: 10.1016/j.bpj.2020.06.007 PMC7376090 32610090

[76] Arlauckas SP Browning EA Poptani H Delikatny EJ. Imaging of cancer lipid metabolism in response to therapy. NMR BioMed. (2019) 32:e4070. doi: 10.1002/nbm.4070 31107583

[77] Fung AA Hoang K Zha H Chen D Zhang W Shi L. Imaging sub-cellular methionine and insulin interplay in triple negative breast cancer lipid droplet metabolism. Front Oncol. (2022) 12:858017. doi: 10.3389/fonc.2022.858017 35359364 PMC8960266

[78] Swartzlander M Bagheri P Hao J Zha H Fung A Shi L. Optical detection of triple negative breast cancer metabolism. International Society for Optical Engineering. Proc. SPIE 11636, Optical Biopsy XIX: Toward Real-Time Spectroscopic Imaging and Diagnosis. (2021) 116360L. doi: 10.1117/12.2585411

[79] Li Z Nguyen C Jang H Hoang D Min S Ackerstaff E. Multimodal imaging of metabolic activities for distinguishing subtypes of breast cancer. BioMed Opt Express. (2023) 14:5764–80. doi: 10.1364/BOE.500252 PMC10659775 38021123

